# Application of Lipidomics in Psychiatry: Plasma-Based Potential Biomarkers in Schizophrenia and Bipolar Disorder

**DOI:** 10.3390/metabo13050600

**Published:** 2023-04-27

**Authors:** Alana C. Costa, Larissa B. Riça, Martinus van de Bilt, Flávia S. Zandonadi, Wagner F. Gattaz, Leda L. Talib, Alessandra Sussulini

**Affiliations:** 1 Laboratory of Neuroscience (LIM-27), Department and Institute of Psychiatry, University of Sao Paulo Medical School, Sao Paulo 05403903, Brazil; alanacosta@usp.br (A.C.C.); gattaz@usp.br (W.F.G.); ledatalib@usp.br (L.L.T.); 2 Instituto Nacional de Biomarcadores em Neuropsiquiatria (INBioN), Conselho Nacional de Desenvolvimento Científico e Tecnológico, Sao Paulo 05403903, Brazil; 3 Laboratory of Bioanalytics and Integrated Omics (LaBIOmics), Institute of Chemistry, University of Campinas (UNICAMP), Campinas 13083970, Brazil; l262803@dac.unicamp.br (L.B.R.); flazando@unicamp.br (F.S.Z.); 4 Instituto Nacional de Ciência e Tecnologia de Bioanalítica (INCTBio), Institute of Chemistry, University of Campinas (UNICAMP), Campinas 13083970, Brazil

**Keywords:** bipolar disorder, biomarkers, lipidomics, plasma, schizophrenia

## Abstract

In this study, we obtained a lipidomic profile of plasma samples from drug-naïve patients with schizophrenia (SZ) and bipolar disorder (BD) in comparison to healthy controls. The sample cohort consisted of 30 BD and 30 SZ patients and 30 control individuals. An untargeted lipidomics strategy using liquid chromatography coupled with high-resolution mass spectrometry was employed to obtain the lipid profiles. Data were preprocessed, then univariate (*t*-test) and multivariate (principal component analysis and orthogonal partial least squares discriminant analysis) statistical tools were applied to select differential lipids, which were putatively identified. Afterward, multivariate receiver operating characteristic tests were performed, and metabolic pathway networks were constructed, considering the differential lipids. Our results demonstrate alterations in distinct lipid pathways, especially in glycerophospholipids, sphingolipids and glycerolipids, between SZ and BD patients. The results obtained in this study may serve as a basis for differential diagnosis, which is crucial for effective treatment and improving the quality of life of patients with psychotic disorders.

## 1. Introduction

Bipolar disorder (BD) is a severe psychiatric illness characterized by alternating mood states of mania, hypomania, depression and/or major depression, as well as mixed states [1]. BD affects more than 1% of the global population, irrespective of country of origin, ethnicity, or level of socioeconomic development [2,3]. The diagnosis of the disease is carried out, essentially, through a directed interview with the patient and family members based on the DSM-5 [4] and the International Statistical Classification of Diseases and Related Health Problems (ICD) [5]. At the beginning of the disease, most patients with BD have a depressive episode that subtly differs from unipolar depression [6], which makes the differential diagnosis very difficult and subjective. Only a small portion of patients with BD in a depressive state are correctly diagnosed during the first year and seek treatment, which can lead to disease progression and patient disability [1,2,7].

Schizophrenia (SZ) is a very severe, complex and disabling psychiatric illness characterized by profound cognitive, behavioral and emotional dysfunctions [8,9]. SZ affects about 1% of the world population, affecting men and women in a similar way [4]. No differences were found in the prevalence of the disease related to ethnicity [10]. The diagnosis of SZ depends on the correct interpretation of the patient report; however, due to the disease heterogeneous symptoms presentation and occurrence, differential diagnosis can be challenging [4,11].

Several studies employing the lipidomics strategy suggested a strong influence of lipid metabolism in the mechanism of BD and SZ [12,13,14,15,16,17]. As an example, it has been observed that certain abnormalities in phospholipid metabolism are strongly associated with SZ [18,19] and that the concentration of omega-3 polyunsaturated fatty acids is lower in individuals with SZ and BD, suggesting potential therapeutic approaches [18,20,21]. It is important to mention that currently, there are no biomarkers that have been approved for the diagnosis of any mental disorder [22,23,24]. Because omic sciences employ a comprehensive approach to the analysis of biomolecules that compose a biological sample, carrying out such studies on these psychiatric diseases is of great importance, especially for the discovery of potential biomarkers that aim at the development of new diagnostic tools [25,26].

Considering the above-mentioned information, the objective of the present study was to perform an untargeted lipidomics analysis of blood plasma samples from healthy individuals and drug-naïve BD and SZ patients in order to examine their lipid profiles and to analyze the correlation of statistically differential lipids with the diseases. These results can contribute toward the identification of potential biomarkers and may help further studies on the diagnosis of BD and SZ and the development of novel treatments.

## 2. Material and Methods

### 2.1. Sample and Clinical Assessments

This open-label study was conducted at the Institute of Psychiatry, University of Sao Paulo, Brazil. The sample consisted of 60 drug-naïve patients (30 SZ and 30 BD) and 30 healthy controls (CT). All participants were under 60 years old and were middle-income, community-dwelling subjects from the hospital catchment area. SZ diagnosis was established according to the Diagnostic and Statistical Manual of Mental Disorders (DSM-IV) [27], and SCID-I/P-Structured Clinical Interview Disorders Axis I for DSM-IV version 2.0 [28] was used to confirm the diagnosis. Psychopathology was assessed using the Positive and Negative Symptoms Scale (PANSS) [29], including the positive and negative subscales and general psychopathology. Depressive and manic symptoms were assessed for BD patients by the Hamilton Depression Rating Scale (HAM-D) [30] and Young’s Mania Rating Scale (YMRS) [31]. Subjects with other psychiatric or neurological disorders were excluded.

The sociodemographic characteristics of the patients and controls are summarized in Table 1.

### 2.2. Chemicals

High-purity methyl-tert-butyl-ether (MTBE), methanol and ammonium acetate were purchased from Sigma Aldrich (Steinheim, Germany). LC-MS grade acetonitrile (ACN), isopropanol (IPA), ammonium formate and formic acid were also purchased from Sigma Aldrich.

### 2.3. Sample Collection and Preparation

Blood samples of all subjects were collected in EDTA-coated tubes (BD Vacutainer^®^, Becton Dickinson, Franklin Lakes, NJ, USA) for plasma metabolite determination after 8 h of fasting. All samples were immediately centrifuged at 20 °C at 1800× *g* for 15 min. The plasma was transferred to a new tube and stored at −80 °C until further processing without previous freeze–thawing.

Plasma lipids were extracted using the SIMPLEX method [32]. Briefly, the plasma samples were incubated with cold methanol and MTBE, followed by the addition of a 0.1% (*m*/*v*) ammonium acetate solution to induce phase separation. The supernatant containing lipids was collected, and the solvents were removed with a vacuum concentrator (Eppendorf, Hamburg, Germany) in a vacuum-high vapor (V-HV) mode at 30 °C for 2 h. Microtubes with the extracted lipids were stored at −80 °C prior to lipidomics analysis. The sample collection and preparation procedures were identical for all samples.

### 2.4. Lipidomics Analysis

Untargeted lipidomics analyses using ultra-high performance liquid chromatography coupled to mass spectrometry (UHPLC-MS) were performed on an UltiMate 3000 UHPLC system (Thermo Fisher Scientific, Waltham, MA, USA) coupled to a QExactive Orbitrap mass spectrometer (Thermo Fisher Scientific). The chromatographic separation was conducted on an ACQUITY CSH C18 (2.1 × 100 mm, 1.7 μm) column (Waters, Milford, MA, USA), and the temperature of the column oven was set to 55 °C. The mobile phase A consisted of an ACN:water mixture (60:40) with 1 mmol/L ammonium formate and 0.1% (*v*/*v*) formic acid, and the mobile phase B consisted of an IPA:ACN mixture (90:10) with 1 mmol/L ammonium formate and 0.1% (*v*/*v*) formic acid. The flow rate was set to 0.4 mL/min, and the injection volume was 5 μL for each sample. The gradient elution program consisted of a first linear gradient from solvent (A/B: 60/40) to solvent (A/B: 57/43) over 2 min; a rapid increase in the solvent (A/B: 50/50); a second linear gradient to the solvent (A/B: 46/54) over 10 min; a rapid increase to solvent (A/B: 30/70); a third linear gradient to solvent (A/B: 1/99) over 6 min; a rapid decrease to solvent (A/B: 60/40); and finally, an isocratic elution of the solvent (A/B: 60/40) for 2 min. The column was equilibrated with solvent (A/B: 60/40) for 5 min before reuse. The total run time was 25 min for each analysis.

MS scans were acquired in the electrospray ionization (ESI) negative mode from *m*/*z* 100 to 1500 with a resolution of 70,000. MS target values and maximum injection time were 3 × 10^6^ ions and 200 ms, respectively. Samples were randomized prior to analysis to reduce the impact of small variations in instrument sensitivity during the measurements. In order to verify system stability, quality control (QC) samples were injected at the start and at the end of a run, and after every 10th sample.

### 2.5. Data Processing and Statistical Analysis

The .raw files were converted to .mzML with MSConvert software (ProteoWizard, Palo Alto, CA, USA). Using RStudio (RStudio, Boston, MA, USA), the parameters for data processing with the ‘xcms’ package [33] were optimized with the ‘IPO’ package [34] based on QC samples. With the optimized parameters, all the files were processed with ‘xcms’ and, afterward, the data were organized in a peak list with the ‘CAMERA’ package [35], followed by the median fold change normalization [36].

Statistical analysis was carried out in the online platform MetaboAnalyst 5.0 (https://www.metaboanalyst.ca; accessed on 24 January 2022) [37]. The resulting peak list was checked for missing values, and features were filtered if their relative standard deviation (RSD) were higher than 20% in QC samples. The filtered dataset was submitted to logarithmic transformation and Pareto scaling, followed by uni- and multivariate analyses. The univariate analysis consisted of a *t*-test, and the multivariate analyses consisted of principal component analysis (PCA) and orthogonal partial least squares discriminant analysis (OPLS-DA).

The differential features indicated by both uni- and multivariate analyses were putatively identified with the CEU Mass Mediator platform (http://ceumass.eps.uspceu.es/index.xhtml; accessed on 19 September 2022) [38,39] based on the experimental mass-to-charge ratios (*m*/*z*), considering HCOO^−^ to be a modifier and [M−H]^−^, [M+Cl]^−^, [M+HCOOH−H]^−^, [M−H−H_2_O]^−^, [M−H+HCOONa]^−^, [2M−H]^−^, [3M−H]^−^, [M−2H]^2−^, [M−3H]^3−^ and [2M+HCOOH−H]^−^ as the potential adducts, with a 10 ppm tolerance. The metabolites were filtered to show only lipids, and all the databases available, except MINE (database composed only of in silico compounds), were used.

Multivariate receiver operating characteristic (ROC) tests were performed in MetaboAnalyst 5.0 with the putatively annotated lipids for each pairwise comparison to ensure that the identified lipids could be considered potential biomarkers (features that did not obtain a match in the putative identification were excluded). MetaboAnalyst 5.0 used Monte-Carlo cross-validation (MCCV) and balanced sub-sampling to generate ROC curves.

Metabolic pathway analysis was performed in Cytoscape [40] using the MetScape plugin [41] and the Correlation Calculator Java application [42]. In order to be able to obtain more comprehensive pathway networks, it was necessary to use more general names for some of the putatively identified lipids when a KEGG compound entry was not available for the specific name (e.g., “sphingomyelin” instead of “sphingomyelin 41:1;O2” or “SM 41:1;O2”). Analogous to ROC tests, features that did not obtain a match in the putative identification were not included. The lipidomics datasets supporting the conclusions of this article were deposited and processed at Metabolomics Workbench [43], with the identifier PR001646 (Study ID ST002554).

## 3. Results

### 3.1. Selection of Potential Lipid Biomarkers

**Figure 1:**
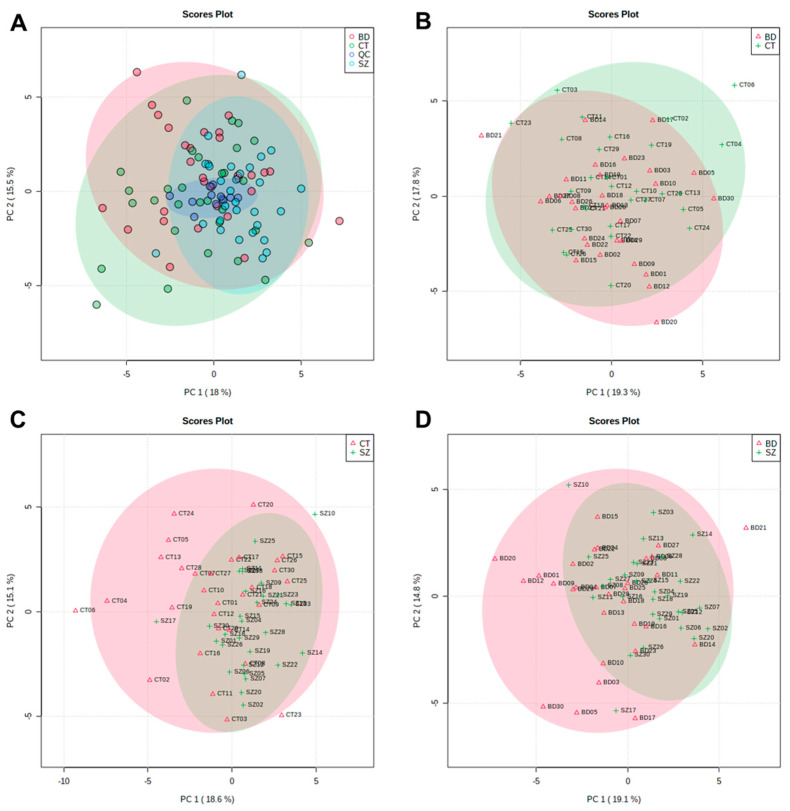
PC1 vs. PC2 scores plot from the PCA of: (**A**) CT, BD, and SZ groups with QCs; (**B**) BD×CT, (**C**) SZ×CT, and (**D**) BD×SZ.

The PCA scores plot that included BD, SZ and CT groups and QC samples are displayed in Figure 1A. QC samples (dark blue) clustered together around the origin, demonstrating that the quality of UHPLC-MS data for this study was satisfactory. Figure 1B–D presents the PCA score plots for BD×CT, SZ×CT, and BD×SZ, respectively. It is noticeable that there was no clear group separation for the three paired comparisons. Despite the overlapping of the three studied groups, SZ group samples were less dispersed than BD and CT (Figure 1A,C,D), presenting a higher similarity among the samples.

Most of the samples scattered in Figure 1 fell into the 95% confidence interval. Samples that fell out of the 95% confidence interval were deemed potential outliers and had their influence on the quality of the analysis evaluated. Whereas neither the PCA clustering nor the OPLS-DA models performance improved considerably with the removal of these samples, they were kept so as not to influence the sample size.

Figures S1–S3 present graphs of the data normalization results, where it was possible to observe that the data distributions were not normal, even after data pretreatment. For this reason, a non-parametric *t*-test (Wilcoxon test) was performed for all three group paired comparisons (BD×CT, SZ×CT, and BD×SZ), considering a *p*-value < 0.05. For BD×CT, no significant features were identified, while for SZ×CT, the Wilcoxon test indicated 49 significant features, and for BD×SZ, 28 significant features were identified. The tables containing the significant features identified by the univariate analysis can be found in the Supplementary Material (Tables S1 and S2).

**Figure 2:**
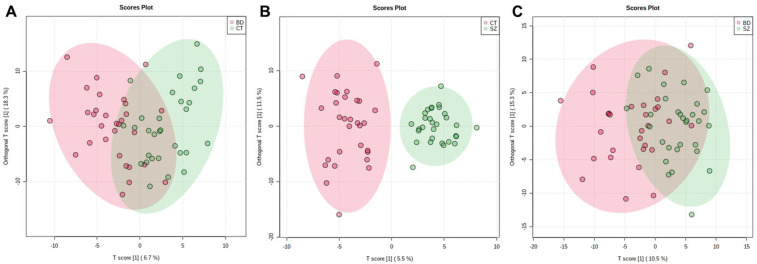
OPLS-DA scores plot for the comparisons between: (**A**) BD×CT, (**B**) SZ×CT, and (**C**) BD×SZ.

The supervised models were also used to investigate if there were significant features that differentiated the groups, which were conducted considering the VIP scores, deeming significant features with a VIP score > 1.0. The OPLS-DA scores plot presented clustering between the SZ and CT groups (Figure 2B). For the other two comparisons, a better clustering tendency was observed between the BD and CT groups (Figure 2A) than between BD and SZ (Figure 2C), and overall, they presented less overlapping when compared to the PCA score plots (Figure 1B–D).

Considering the predictive and orthogonal components, respectively, the OPLS-DA indicated: 139 and 154 significant features for the analysis between BD×CT (Table S3), 121 and 196 significant features for SZ×CT (Table S4), and 130 and 159 significant features for BD×SZ (Table S5). Because the univariate analysis did not identify any significant features for BD×CT, the feature selection for this comparison occurred only with those identified by OPLS-DA, totaling 116 features, which were described in Table S6 with their respective putative identifications. Between SZ×CT, 49 features were selected by both univariate and multivariate analyses, which can be found in Table S7, along with their putative identifications. Table S8 contains 28 features selected by uni- and multivariate analyses of the BD×SZ comparison and their putative identifications. Table 2 displays the prediction parameters for the OPLS-DA models. Although the SZ×CT OPLS-DA model presented a superior performance than the others, they performed poorly overall as prediction models.

**Figure 3:**
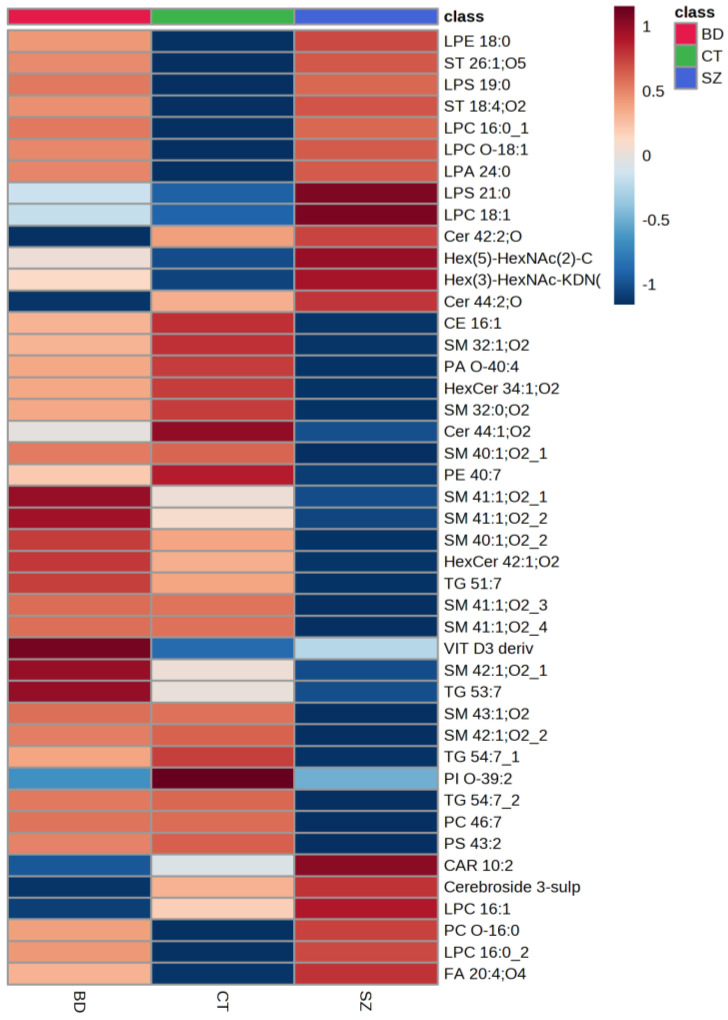
Comparison of the main lipid species across the studied groups. The heatmap presents the average intensities of important lipid features according to ANOVA compared with the studied groups.

Figure 3 presents a heatmap of the comparison of the main lipid species across the studied groups, i.e., the lipids common among the three pairwise comparisons. Generally, the SZ group presented lower relative concentrations for most of the lipid species when compared to BD and CT. When evaluating the lipid species with a higher mean intensity in SZ than in the CT group, we observed that glycerophospholipids (GP), particularly LPC 16:0, sphingolipids (SP), such as glycosphingolipids and ceramides, several types of sterol lipids (ST), and fatty acids (FA) were on the top of the list. When examining those with a lower average intensity, most of the lipid species were SP, predominantly sphingomyelins; glycerolipids (GL), such as triacylglycerols, as well as some species of GP and ST. By contrast, SP presented lower average intensities for the BD group when compared to CT, alongside some FA species. Oppositely to SZ, the BD group presented higher average intensities for GL, such as triacylglycerides, and SP, mostly sphingomyelins, when compared to the CT group, as well as some species of GP, FA, and ST, such as a vitamin D3 derivative.

### 3.2. Differential Lipids and Potentially Altered Biochemical Pathways

**Figure 4:**
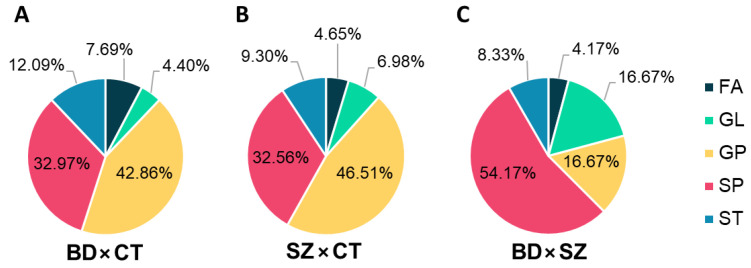
Distribution of lipid classes in the differential lipids identified for (**A**) BD×CT, (**B**) SZ×CT, and (**C**) BD×SZ comparisons. FA, fatty acyls; GL, glycerolipids; GP, glycerophospholipids; SP, sphingolipids; ST, sterol lipids.

Differential lipids (DLs), i.e., the differential features that held a putative annotation, which shared the same lipid class for each comparison, were grouped and plotted on the graphs displayed in Figure 4. GP and SP made up most of the DLs between BD and CT groups (Figure 4A), followed by ST, FA, and GL. With a slightly higher fraction of GL, the DLs of the SZ×CT comparison followed a similar pattern (Figure 4B). When comparing BD×SZ, however, DLs were mostly SP (Figure 4C), followed by GL and GP at equal proportions, and ST and FA in lower amounts.

**Figure 5:**
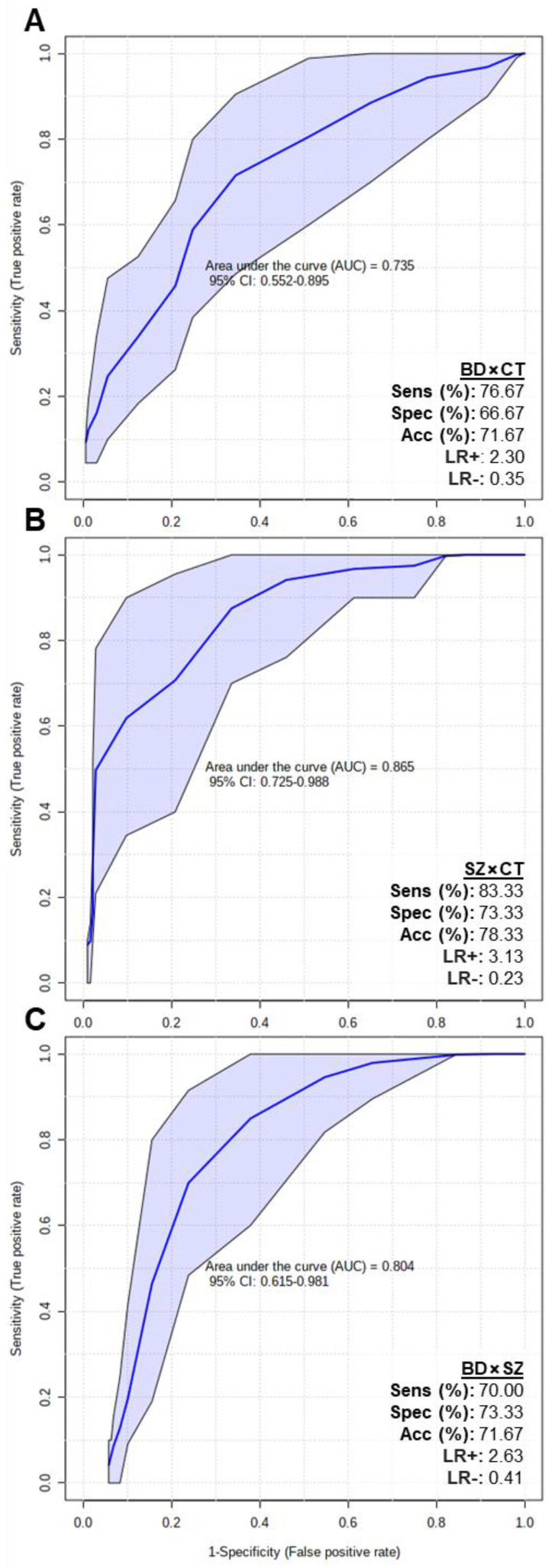
Multivariate ROC test curves and figures of merit for (**A**) BD×CT, (**B**) SZ×CT, and (**C**) BD×SZ models. The ROC test classification method was PLS-DA and feature ranking method was PLS-DA built-in with 1 latent variable. The models were built with all the differential putatively identified lipids of each comparison: (**A**) BD×CT, 90 lipid species; (**B**) SZ×CT, 44 lipid species; and (**C**) BD×SZ, 23 lipid species. CI, confidence interval; Sens, sensitivity; Spec, specificity; Acc, accuracy; LR+, positive likelihood ratio; LR-, negative likelihood ratio.

In order to assess if the putatively identified lipids could be considered potential biomarkers, multivariate ROC tests were performed, and the results are displayed in Figure 5. The BD×CT model (Figure 5A) presented an area under the curve (AUC) of 0.735 (0.552–0.895 with 95% confidence interval), 76.67% of sensitivity (Sens), 66.67% of specificity (Spec) and 71.67% of accuracy (Acc). SZ×CT model (Figure 5B) presented AUC = 0.865 (0.725–0.988 with 95% confidence interval), Sens = 83.33%, Spec = 73.33% and Acc = 78.33%. BD×SZ model (Figure 5C) presented AUC = 0.804 (0.615–0.981 with 95% confidence interval), Sens = 70%, Spec = 73.33% and Acc = 71.67%. Overall, all 3 ROC curves presented good AUC values (close to 1.000), with the SZ×CT model reaching 0.988 at the 95% confidence interval. The models also presented adequate Sens, Spec and Acc parameters.

**Figure 6:**
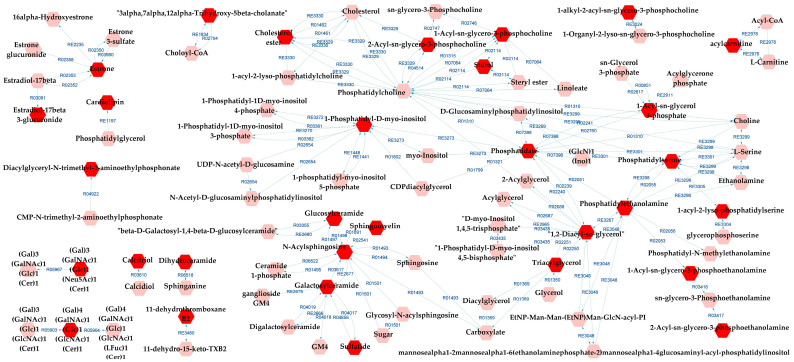
Differential lipids (DLs)-based metabolic network linked with potentially altered metabolic pathways between BD and CT groups. The DLs are indicated by the bright red hexagons. Compounds in the pale red hexagons link the DLs with each other and/or show their relationship with a biochemical pathway to build the metabolic network.

Figure 6 presents the metabolic network that connects the potentially altered pathways to the DLs, providing a visual representation of these results. According to the metabolic pathways analysis of the DLs for BD×CT, the following pathways were possibly affected: androgen and estrogen biosynthesis and metabolism, bile acid biosynthesis, GP metabolism, glycosphingolipid (GSP) biosynthesis—globo series, GSP metabolism, linoleate metabolism, phosphatidylinositol phosphate metabolism, prostaglandin formation from arachidonate, and vitamin D3 metabolism. The reaction modules tables used to build the metabolic network in Figure 6 can be found in Tables S9 and S10.

**Figure 7:**
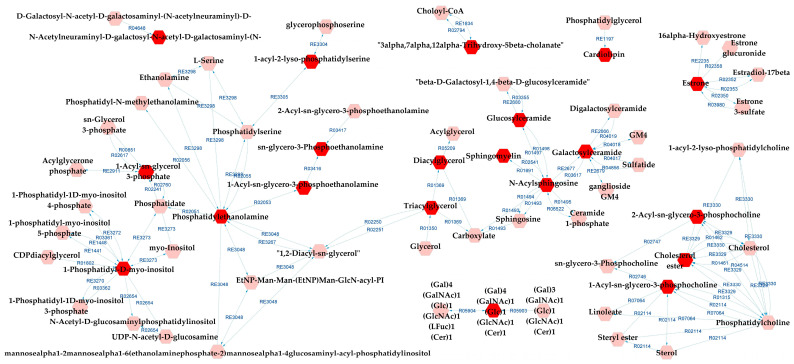
Differential lipids (DLs)-based metabolic network linked with potentially altered metabolic pathways between SZ and CT groups. The DLs are indicated by the bright red hexagons. Compounds in the pale red hexagons link the DLs with each other and/or show their relationship with a biochemical pathway to build the metabolic network.

Metabolic pathways analysis for SZ×CT revealed that most of the possibly altered pathways were identical to those for BD×CT, exceptions being prostaglandin formation from arachidonate and vitamin D3 metabolism (which did not appear), and GSP biosynthesis—ganglio series (that was not present for BD×CT). Similarly, Figure 7 presents the metabolic network resulting from the analysis. Tables S11 and S12 present the reaction modules tables used to build the metabolic network presented in Figure 7.

**Figure 8:**
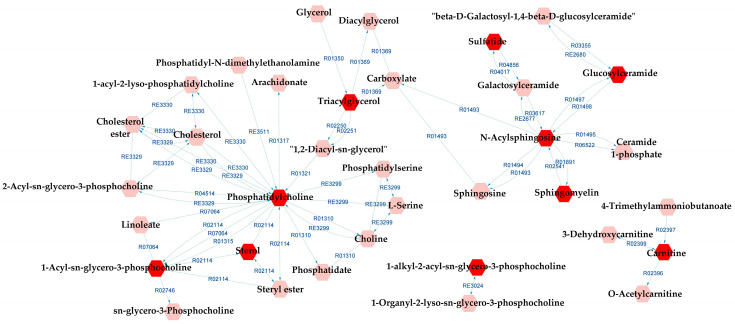
Differential lipids (DLs)-based metabolic network linked with potentially altered metabolic pathways between BD and SZ groups. The DLs are indicated by the bright red hexagons. Compounds in the pale red hexagons link the DLs with each other and/or show their relationship with a biochemical pathway to build the metabolic network.

When comparing BD and SZ groups, GP metabolism, GSP metabolism, and linoleate metabolism also appeared as potentially affected metabolic pathways in relation to the DLs. In addition to them, arachidonic acid metabolism, lysine metabolism, and urea cycle and metabolism of arginine, proline, glutamate, aspartate, and asparagine were indicated by the metabolic pathways analysis. A complete metabolic network comprising the aforementioned pathways alongside the DLs is presented in Figure 8, and the reaction modules tables used to build the metabolic network displayed in Figure 8 can be found in Tables S13 and S14.

## 4. Discussion

In this study, our objective was to perform an untargeted lipidomics analysis of blood plasma samples from healthy individuals and drug-naïve BD and SZ patients. Our results of AUC values, alongside other metric figures, suggest that the putatively identified lipids performed well in differentiating the groups pairwise and, therefore, could function as potential biomarkers for BD and SZ. These results support the hypothesis that lipid alterations may be part of the core of changes in the central nervous system that occur in these disorders.

Since all psychiatric patients included in the study were drug-free and most likely early in their disease course, metabolic differences may not have been sufficiently pronounced to be detected by unsupervised multivariate statistical analysis. Despite this, our results demonstrate changes in different lipid pathways, such as glycerophospholipids, glycerolipids, and sphingolipids. Some metabolic pathways stood out in the differentiation of the studied groups. For example, vitamin D3 metabolism and prostaglandin formation from arachidonate were only altered when comparing BD and CT groups.

Plasma metabolites were increasingly investigated as potential biomarkers in psychiatry, offering a way to objectively measure the biochemical changes underlying mental disorders. Recent studies have suggested that certain plasma metabolites may be involved in psychiatric conditions such as depression [44], anxiety [45], bipolar disorder [46], and schizophrenia [47]. These metabolites can be used both in the early identification of psychiatric disorders and in monitoring the effectiveness of treatments, allowing for the personalized therapy of each patient. However, the use of plasma metabolites as biomarkers still faces challenges, such as the heterogeneity of psychiatric disorders and the lack of large-scale validation. The results presented herein help to fill the knowledge gap in the scientific literature by shedding light on some metabolic pathways that may differentiate psychiatric disorders in plasma.

It is believed that glycerophospholipid dysfunction is associated with changes in the activity of the enzyme phospholipase A_2_ (PLA_2_), which is responsible for breaking down glycerophospholipids into fatty acids and other lipids. It has been demonstrated that PLA_2_ activity is increased in SZ and BD and has an ultra-high risk (UHR) for psychosis patients [48,49,50,51,52,53]. In fact, studies have demonstrated a decrease in levels of phosphatidylcholine and sphingomyelin in the brain tissue of SZ patients, including the prefrontal cortex and hippocampus. These alterations appear to be related to changes in neuronal function and may be associated with symptoms of cognitive deficit and alterations in sensory perception [54,55,56]. Our results corroborate these findings since the SZ group presented a decrease in most lipid species, suggesting an increase in phospholipase metabolism in these patients. In BD, a decrease in the levels of phosphatidylethanolamine in the prefrontal cortex and amygdala and an increase in levels of phosphatidylcholine in the prefrontal cortex have been reported. These alterations also appear to be associated with changes in neuronal function and may contribute to affective and cognitive symptoms [57]. Similar results were not found in this study, which may be justified by our sample consisting of patients in the early stages of the disease.

Moreover, in line with our results, some studies found that metabolite levels related to the kynurenine pathway—marked by an increase in the kynurenine and a decrease in the production of the quinoline, which is involved in the metabolism of tryptophan—are different between SZ and BD [58,59]. Additionally, Lin et al. reported differences in the levels of certain glycerophospholipids in SZ patients plasma [60]. Other factors that can also influence the course of the disease, such as environmental factors, epigenetics and treatment, for example, cannot be ignored. Ribeiro et al. demonstrated that treated BD patients exhibited significant alterations in the levels of proteins and metabolites, which were related to blood coagulation and platelet function, suggesting that disturbances in the hemostatic system may be involved in the pathogenesis of BD [61]. In another study, it was demonstrated that treated patients with BD type I exhibited significant alterations in the levels of various lipids, including free fatty acids, ceramides, and phospholipids. Additionally, significant differences were found in the expression of certain genes involved in the synthesis and metabolism of lipids [62]. Therefore, all these findings suggest that lipid alterations were unquestionable, both in SZ and in BD.

The differential diagnosis is crucial in psychotic disorders, such as SZ and BD, because these diseases can share similar symptoms, such as hallucinations and delusions, but have different causes and treatments. An accurate diagnosis is essential for choosing the appropriate treatment and improving the quality of life of patients. The treatment of SZ usually involves antipsychotics, while the treatment of BD may involve mood stabilizers, antipsychotics, or antidepressants. These treatments may have different side effects and may be effective depending on the correct diagnosis. In addition, SZ and BD have different patterns of evolution and prognosis, which can influence treatment choices and the therapeutic approach. Therefore, it is essential to establish biomarkers that can differentiate these psychotic disorders.

Plasma lipids are a promising potential diagnostic tool for differentiating SZ and BD; however, this is still not a definitive and completely validated method and should be used in conjunction with other clinical assessments and diagnostic criteria. A limitation of this pioneer and exploratory work is that differential lipids were putatively identified. Hence, further studies employing a targeted lipidomics strategy are required to confirm their identities and validate the differential lipid profile of SZ and BD.

## 5. Conclusions

While the application of lipidomics in psychiatric disorders is still in its early stages, the results reported in this study suggest that alterations mainly in glycerophospholipids and sphingolipids levels may play an important role in the pathogenesis of schizophrenia and bipolar disorder. The identification of these metabolic alterations may aid in the development of new therapeutic approaches for the treatment of these complex mental disorders, in addition to the development of novel diagnostic tools. Nonetheless, validation through targeted lipidomics and further research are necessary to fully understand the implications of these metabolic alterations and how they can be used in the diagnosis and treatment of psychotic disorders.

## Figures and Tables

**Table 1:** Socio-demographic characteristics of patients and controls included in the study.

Characteristic	SZ (*n* = 30)	BD (*n* = 30)	CT (*n* = 30)	*p* ^†^
Gender (M/F)	16/14	11/19	15/15	-
Age (mean ± SD)	26.5 ± 6.8	26.6 ± 4.4	26.5 ± 2.2	0.999
Education (mean ± SD)	11.8 ± 3.0	12.6 ± 2.1	14.3 ± 2.8	**0.002**
PANSS (mean ± SD)	78 ± 22	-	-	-
PANSS—Positive symptoms (mean ± SD)	19 ± 5	-	-	-
PANSS—Negative symptoms (mean ± SD)	18 ± 8	-	-	-
HAM-D (mean ± SD)	-	15 ± 8	-	-
YMRS (mean ± SD)	-	9 ± 8	-	-

**Legend:** SZ = schizophrenia; BD = bipolar disorder; CT = healthy controls; M = male; F = female; SD = standard deviation; PANSS = Positive and Negative Syndrome Scale; HAM-D = Hamilton Depression Rating Scale; YMRS = Young Mania Rating Scale. ^†^ One-way analysis of variance (one-way ANOVA) was performed for age and education. Bold values indicate significant *p*-values (*p* < 0.05).

**Table 2:** OPLS-DA prediction parameters. R^2^X and R^2^Y represent the explained sum of squares for X and Y of the OPLS-DA model and Q^2^ indicates the prediction ability of the model.

Group Comparison		R^2^X	R^2^Y	Q^2^
BD×CT	p1	0.061	0.259	0.020
	o1	0.176	0.310	0.045
SZ×CT	p1	0.064	0.415	0.292
	o1	0.127	0.299	0.169
BD×SZ	p1	0.106	0.253	0.052
	o1	0.148	0.183	−0.054

p1, predictive component; o1, orthogonal component.

## Data Availability

The data presented in this study are available in Metabolomics Workbench at http://dx.doi.org/10.21228/M8XX43 (accessed on 30 March 2023), reference number PR001646 (Study ID ST002554).

## Supplementary Materials

The following supporting information can be downloaded at: https://www.mdpi.com/article/10.3390/metabo13050600/s1, Figure S1: Data distribution before and after normalization for variables (top, referred as features) and samples (bottom) regarding the BD×CT comparison; Figure S2: Data distribution before and after normalization for variables (top, referred as features) and samples (bottom) regarding the SZ×CT comparison; Figure S3: Data distribution before and after normalization for variables (top, referred to as features) and samples (bottom) regarding the BD×SZ comparison. Table S1: List of significant features identified by the Wilcoxon test between SZ×CT, from lowest to highest *p*-value; Table S2: List of significant features identified by the Wilcoxon test between BD×SZ, from lowest to highest *p*-value; Table S3: List of significant features identified by OPLS-DA between BD and CT groups, in descending order of VIP scores; Table S4: List of significant features identified by OPLS-DA between CT and SZ groups, in descending order of VIP scores; Table S5: List of significant features identified by OPLS-DA between BD and SZ groups, in descending order of VIP scores; Table S6: Mass spectrometry characterization with theorical (Th) and experimental (Exp) masses with the *m*/*z* and putative identification for the significant features of the BD×CT comparison; Table S7: Mass spectrometry characterization with theorical (Th) and experimental (Exp) masses with the *m*/*z* and putative identification for the significant features of the SZ×CT comparison; Table S8: Mass spectrometry characterization with theorical (Th) and experimental (Exp) masses with the *m*/*z* and putative identification for the significant features of the BD×SZ comparison; Table S9: MetScape node table from the metabolic pathway analysis of the BD×CT comparison (related to Figure 6); Table S10: MetScape edge table from the metabolic pathway analysis of the BD×CT comparison (related to Figure 6); Table S11: MetScape node table from the metabolic pathway analysis of the SZ×CT comparison (related to Figure 7); Table S12: MetScape edge table from the metabolic pathway analysis of the SZ×CT comparison (related to Figure 7); Table S13: MetScape node table from the metabolic pathway analysis of the BD×SZ comparison (related to Figure 8); Table S14: MetScape edge table from the metabolic pathway analysis of the BD×SZ comparison (related to Figure 8).
