# Supplementary material for: Application of Lipidomics in Psychiatry: Plasma-Based Potential Biomarkers in Schizophrenia and Bipolar Disorder
Source: Metabolites. 2023 Apr 27;13(5):600. doi: 10.3390/metabo13050600 (PMC10222421; doi:10.3390/metabo13050600)
Supplement: Supplementary file 1 [file metabolites-13-00600-s001.zip › metabolites-2347063-supplementary.pdf]

Supplementary Material

Application of Lipidomics in Psychiatry: Plasma-Based Potential Biomarkers in Schizophrenia and Bipolar Disorder

Alana C. Costa [1,2,†], Larissa B. Riça [3,†], Martinus van de Bilt [1,2], Flávia S. Zandonadi [3], Wagner F. Gattaz [1,2], Leda L. Talib [1,2,†] and Alessandra Sussulini [3,4,*,†]

[1] Laboratory of Neuroscience (LIM-27), Department and Institute of Psychiatry, University of Sao Paulo Medical School, Sao Paulo 0540390,, Brazil; alanacosta@usp.br (A.C.C.); martinus.bilt@gmail.com (M.v.d.B.); gattaz@usp.br (W.F.G.); ledatalib@usp.br (L.L.T.)
[2] Instituto Nacional de Biomarcadores em Neuropsiquiatria (INBioN), Conselho Nacional de Desenvolvimento Científico e Tecnológico, Sao Paulo 05403903, Brazil
[3] Laboratory of Bioanalytics and Integrated Omics (LaBIOmics), Institute of Chemistry, University of Campinas (UNICAMP), Campinas 13083970, Brazil; l262803@dac.unicamp.br (L.B.R.); flazando@unicamp.br (F.S.Z.)
[4] Instituto Nacional de Ciência e Tecnologia de Bioanalítica (INCTBio), Institute of Chemistry, University of Campinas (UNICAMP), Campinas 13083970, Brazil
* Correspondence: sussulini@unicamp.br; Tel.: +55-19-3521-3060
† These authors contributed equally to this work.

Figure S1. Data distribution before and after normalization for variables (top, referred as features) and samples (bottom) regarding the BD×CT comparison.

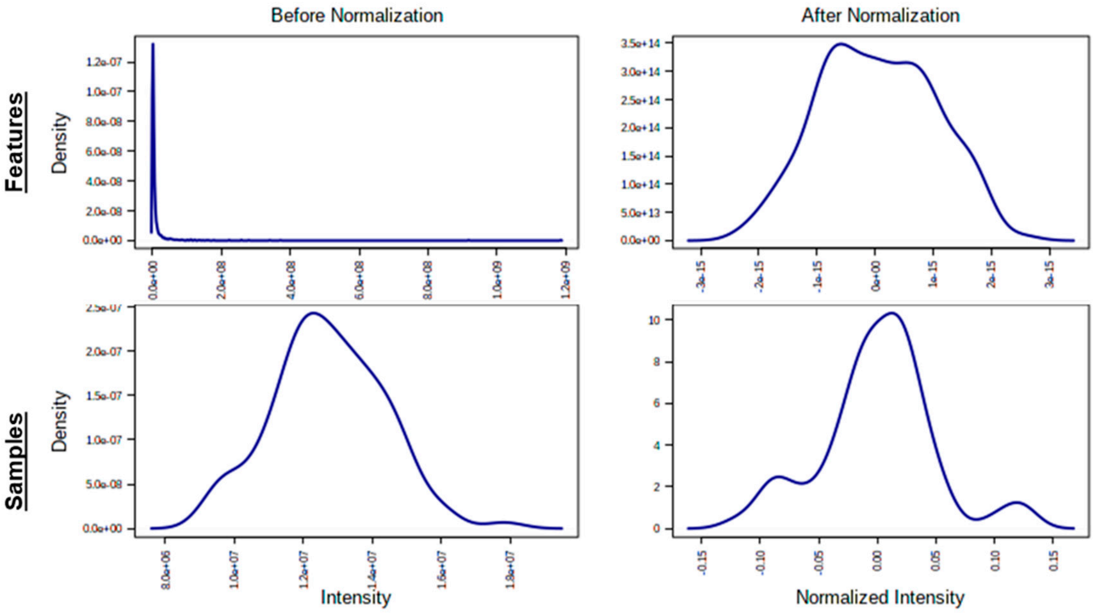

Figure S2. Data distribution before and after normalization for variables (top, referred as features) and samples (bottom) regarding the SZ×CT comparison.

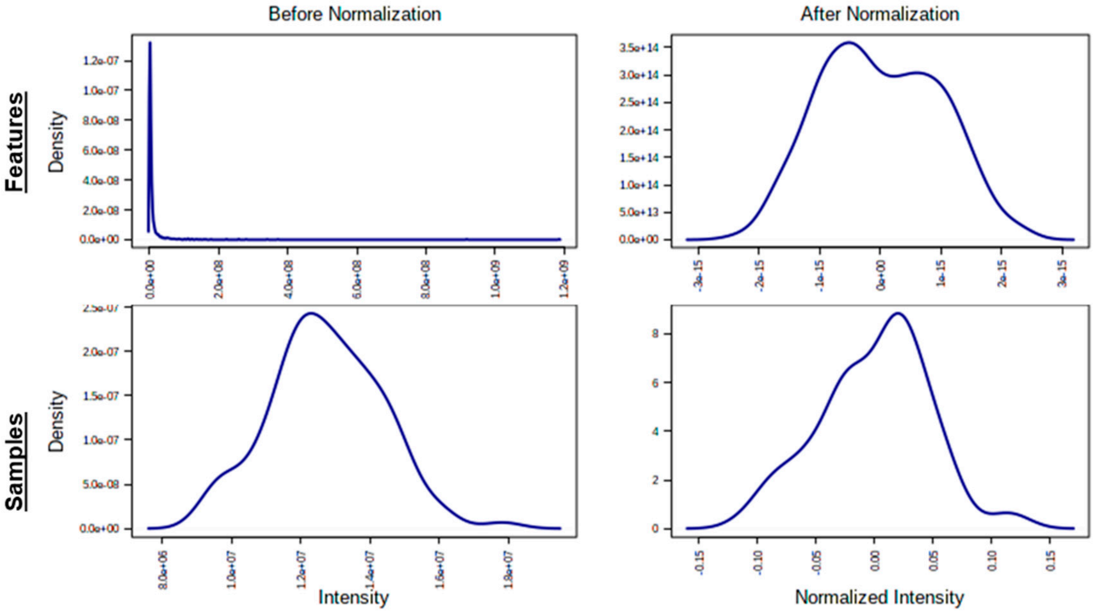

Figure S3. Data distribution before and after normalization for variables (top, referred as features) and samples (bottom) regarding the BD×SZ comparison.

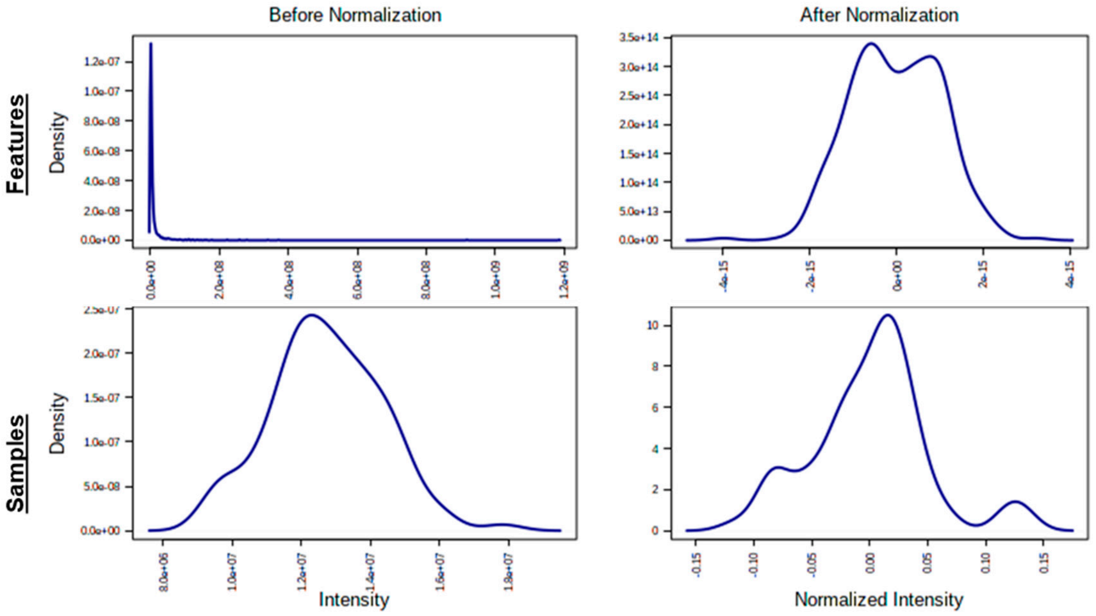

Table S1. List of significant features identified by the Wilcoxon test between SZ×CT, from lowest to highest *p*-value

Feature	*p*-value
621.3399	4.06×10^{-5}
904.6418	5.05×10^{-5}
899.6509	5.83×10^{-5}
620.3373	7.22×10^{-5}
873.7088	7.22×10^{-5}
903.6377	8.30×10^{-5}
785.6548	9.53×10^{-5}
846.6819	1.25×10^{-4}
875.7151	1.43×10^{-4}
845.6788	1.64×10^{-4}
900.6545	1.75×10^{-4}
207.9167	1.87×10^{-4}
874.7135	1.87×10^{-4}
833.6697	2.27×10^{-4}
832.6672	2.42×10^{-4}
673.5293	5.11×10^{-4}
736.6858	5.11×10^{-4}
831.6636	5.43×10^{-4}
913.6670	5.43×10^{-4}
101.5198	7.30×10^{-4}
659.5131	7.74×10^{-4}
914.6698	7.74×10^{-4}
727.6554	8.20×10^{-4}
733.5537	8.20×10^{-4}
720.5408	9.75×10^{-4}
719.5374	1.03×10^{-3}
731.5375	1.03×10^{-3}
721.5444	1.16×10^{-3}
539.3208	1.29×10^{-3}
734.5572	1.37×10^{-3}
538.3174	2.22×10^{-3}
567.3524	2.47×10^{-3}
788.5286	2.60×10^{-3}
867.6409	2.74×10^{-3}
1103.6560	2.74×10^{-3}
1104.6600	3.72×10^{-3}
889.6218	4.31×10^{-3}
566.3490	4.53×10^{-3}
1035.6680	4.53×10^{-3}
1036.6720	4.53×10^{-3}
1037.6760	4.53×10^{-3}
542.3393	4.76×10^{-3}
614.3486	4.76×10^{-3}
930.6524	4.99×10^{-3}
481.3145	5.24×10^{-3}
541.3365	5.24×10^{-3}
480.3114	5.50×10^{-3}
100.0728	6.34×10^{-3}
543.3421	6.34×10^{-3}

Table S2. List of significant features identified by the Wilcoxon test between BD×SZ, from lowest to highest *p*-value

Feature	*p*-value
862.6984	1.99×10^{-4}
832.6672	2.93×10^{-4}
873.7088	2.93×10^{-4}
833.6697	3.54×10^{-4}
984.6292	3.76×10^{-4}
785.6548	4.26×10^{-4}
207.9167	4.53×10^{-4}
831.6636	4.53×10^{-4}
799.6701	5.11×10^{-4}
800.6729	6.49×10^{-4}
845.6788	6.49×10^{-4}
214.9244	7.30×10^{-4}
846.6819	7.30×10^{-4}
860.6979	8.69×10^{-4}
932.6232	8.69×10^{-4}
861.7015	9.21×10^{-4}
859.6946	9.75×10^{-4}
874.7135	9.75×10^{-4}
612.5961	1.44×10^{-3}
875.7151	1.44×10^{-3}
985.6209	1.44×10^{-3}
640.6277	1.70×10^{-3}
913.6670	2.74×10^{-3}
473.4018	2.88×10^{-3}
101.5198	3.19×10^{-3}
914.6698	3.19×10^{-3}
876.6858	3.26×10^{-3}
928.6860	3.53×10^{-3}

Tables S3 to S5. Available as .xls file.

Table S6. Mass spectrometry characterization with theorical (Th) and experimental (Exp) masses with the *m/z* and putative identification for the significant features of the BD×CT comparison

Lipid Class	Putative identification	ID Level†	Adduct	Mass Error (ppm)	*m/z* Exp	*m/z* Th
—	*nf*	4	—	—	101.5198	—
FA	Caproic acid	2	$[M-H]^-$	6	115.0758	116.0837
—	*nf*	4	—	—	138.9330	—
—	*nf*	4	—	—	214.4207	—
—	*nf*	4	—	—	214.9244	—
—	*nf*	4	—	—	237.8966	—
—	*nf*	4	—	—	246.9052	—
—	*nf*	4	—	—	249.9791	—
—	*nf*	4	—	—	262.0156	—
FA	FA 18:0;O2	2	$[M-H]^-$	4	315.2553	316.2614
—	*nf*	4	—	—	367.9082	—
—	*nf*	4	—	—	371.9026	—
—	*nf*	4	—	—	396.8927	—
—	*nf*	4	—	—	433.8786	—
—	*nf*	4	—	—	435.8956	—
—	*nf*	4	—	—	439.8901	—
GP	LPE 16:0	2	$[M-H]^-$	5	452.2804	453.2855
ST	ST 24:1;O5	2	$[M+HCOOH-H]^-$	4	453.2841	408.2876
ST	ST 29:1;O	2	$[M+CH_3COOH-H]^-$	4	473.4018	428.4018
GP	LPE 18:1	2	$[M-H]^-$	4	478.2959	479.3012
ST	ST 25:2;O5	2	$[M+CH_3COOH-H]^-$	5	479.2993	420.2876
GP	LPE 18:0	2	$[M-H]^-$	4	480.3114	481.3168
ST	ST 26:1;O5	2	$[M+HCOOH-H]^-$	6	481.3145	436.3189

GP	LPE 20:4	2	$[M-H]^-$	5	500.2807	501.2855
ST	ST 29:4;O8	2	$[M-H-H_2O]^-$	2	501.2839	520.3036
—	*nf*	4	—	—	507.8775	—
ST	VIT D3 deriv‡	2	$[M+K-2H]^-$	9	527.3191	490.3658
—	*nf*	4	—	—	527.8726	—
GP	LPS 19:0	2	$[M-H]^-$	4	538.3174	539.3223
ST	ST 18:4;O2	2	$[2M-H]^-$	8	539.3208	270.1620
GP	PC 16:0	2	$[M+HCOOH-H]^-$	6	540.3337	495.3325
GP	LPC 16:0	2	$[M+HCOOH-H]^-$	3	541.3365	496.3398
GP	LPC O-18:1	2	$[M+Cl]^-$	2	542.3393	507.3689
GP	LPA 24:0	2	$[M+Na-2H]^-$	2	543.3421	522.3685
GP	LPC 18:3	2	$[M+HCOOH-H]^-$	5	562.3176	517.3168
GP	LPC 16:0	2	$[M-H+HCOONa]^-$	1	563.3211	496.3403
GP	LPS 21:0	2	$[M-H]^-$	5	566.3490	567.3536
GP	LPC 18:1	2	$[M+HCOOH-H]^-$	3	567.3524	522.3560
GP	LPS O-20:0	2	$[M+K-2H]^-$	5	576.3098	539.3587
—	*nf*	4	—	—	585.5682	—
GP	PHOOA-PE	2	$[M-H-H_2O]^-$	5	588.3334	607.3485
GP	LPC 18:1	2	$[M-H+HCOONa]^-$	1	589.3366	522.3560
GP	PA 30:3	2	$[M-H-H_2O]^-$	11	595.3833	614.3948
GP	PHHdiA-PE	2	$[M-H]^-$	2	608.3217	609.3278
—	*nf*	4	—	—	609.3243	—
SP	Monosialyl-Gb5	2	$[M-3H]^{3-}$	16	610.3273	1834.0340
SP	Cer 42:2;O	2	$[M-H-H_2O]^-$	19	612.5961	631.6267
—	*nf*	4	—	—	613.5998	—
SP	Hex(5)-HexNAc(2)-Cer 42:2;O2	2	$[M-3H]^{3-}$	6	620.3373	1864.0445
SP	Hex(3)-HexNAc-KDN(2)-Cer 44:1;O2	2	$[M-3H]^{3-}$	1	621.3399	1867.0442
FA	LTC4	2	$[M-H]^-$	0	624.2958	625.3033
SP	Cer 44:2;O	2	$[M-H-H_2O]^-$	18	640.6277	659.6580
—	*nf*	4	—	—	641.6310	—

—	*nf*	4	—	—	643.8533	—
ST	CE 16:1	2	$[M+K-2H]^-$	7	659.5131	622.5689
SP	Cer 42:1;O	2	$[M+Cl]^-$	10	668.6052	633.6424
SP	SM 32:1;O2	2	$[M-H]^-$	1	673.5293	674.5363
SP	Cer 38:2;O3	2	$[M-H+HCOONa]^-$	1	674.5338	607.5540
GP	LPI 20:5	2	$[M-H+HCOONa]^-$	7	685.2566	618.2805
ST	Glucoronide	2	$[M+Cl]^-$	7	686.2630	651.2891
SP	Cer 42:0;O3	2	$[M+Na-2H]^-$	9	688.6286	667.6479
—	*nf*	4	—	—	697.8190	—
GP	PA O-40:4	2	$[M-H-H_2O]^-$	1	719.5374	738.5563
SP	HexCer 34:1;O2	2	$[M+Na-2H]^-$	2	720.5408	699.5649
SP	SM 32:0;O2	2	$[M+HCOOH-H]^-$	8	721.5444	331.2643
SP	Cer 42:0;O3	2	$[M+CH_3COOH-H]^-$	6	726.6579	667.6479
FA	FAHFA 24:3-(2-O-24:0)	2	$[M-H]^-$	8	727.6554	728.6683
SP	SM 33:2;O2	2	$[M+HCOOH-H]^-$	4	731.5375	686.5363
GL	DG 42:5	2	$[M+Cl]^-$	1	733.5537	698.5849
—	*nf*	4	—	—	734.2657	—
GP	PC O-35:4	2	$[M-H-H_2O]^-$	11	734.5572	753.5672
GP	PA O-38:2	2	$[M+Na-2H]^-$	6	735.5270	714.5563
SP	Cer 44:1;O2	2	$[M+CH_3COOH-H]^-$	5	736.6858	677.6686
SP	SM 34:2;O2	2	$[M+HCOOH-H]^-$	4	745.5531	700.5519
SP	Cerebroside	2	$[M+Na-2H]^-$	2	746.5570	725.5806
GP	PG O-34:1	2	$[M+Na-2H]^-$	10	755.5135	734.5462
SP	SM 40:1;O2	2	$[M-H]^-$	1	785.6548	786.6615
GP	PA 41:4	2	$[M+Na-2H]^-$	0	787.5261	766.5513
GP	PE 40:7	2	$[M-H]^-$	6	788.5286	789.5309
GP	PA O-42:6	2	$[M+Cl]^-$	4	797.5229	762.5563
SP	SM 41:2;O2	2	$[M-H]^-$	1	797.6549	798.6615

SP	SM 41:1;O2	2	$[M-H]^-$	0	799.6701	800.6771
SP	SM 41:1;O2	2	$[M-H]^-$	6	800.6729	801.6850
GP	PE 42:8	2	$[M-H]^-$	8	814.5455	815.5465
—	*nf*	4	—	—	822.8315	—
SP	SM 41:1;O2	2	$[M+HCOOH-H]^-$	4	845.6788	800.6771
SP	SM 41:1;O2	2	$[M+HCOOH-H]^-$	2	846.6819	801.6850
ST	VIT D3 deriv‡	2	$[2M-H]^-$	4	859.6859	430.3447
SP	SM 42:1;O2	2	$[M+HCOOH-H]^-$	4	859.6946	814.6928
SP	SM 42:1;O2	2	$[M+HCOOH-H]^-$	1	860.6979	815.7006
GL	TG 53:7	2	$[M-H]^-$	4	861.7015	862.7050
GL	TG 52:6	2	$[M+Cl]^-$	1	871.6947	836.7258
SP	SM 43:2;O2	2	$[M+HCOOH-H]^-$	2	872.6968	827.7006
SP	SM 43:1;O2	2	$[M+HCOOH-H]^-$	3	873.7088	828.7084
SP	SM 42:1;O2	2	$[M+CH_3COOH-H]^-$	1	874.7135	815.7006
GL	TG 54:7	2	$[M-H]^-$	2	875.7151	876.7207
FA	CAR 18:0;O	2	$[2M-H]^-$	4	885.7110	443.3611
SP	SM 43:2;O2	2	$[M+CH_3COOH-H]^-$	2	886.7129	827.7006
SP	SM 44:1;O2	2	$[M+HCOOH-H]^-$	1	888.7296	843.7319
GP	PI O-39:2	2	$[M-H]^-$	5	889.6218	890.6248
GP	PC O-42:7	2	$[M+HCOOH-H]^-$	1	890.6269	845.6298
GP	PC O-42:3	2	$[M+HCOOH-H]^-$	3	898.6935	853.6924
ST	20:1-Glc-Campesterol	2	$[M+HCOOH-H]^-$	1	899.6976	854.7000
GP	PC O-42:2	2	$[M+HCOOH-H]^-$	4	900.7099	855.7081
GP	PS 43:2	2	$[M+HCOOH-H]^-$	9	930.6524	885.6459
FA	CAR 10:2	2	$[3M-H]^-$	2	932.6232	311.2097
GP	PI O-40:5	2	$[M+CH_3COOH-H]^-$	3	957.6097	898.5935
GP	PI O-42:2	2	$[M+Cl]^-$	3	967.6386	932.6718

GP	PE-NMe2 46:7	2	$[M-H+HCOONa]^-$	8	968.6433	901.6561
SP	Cerebroside 3-sulphate	2	$[M-H+HCOONa]^-$	15	984.6292	917.6626
GP	LPC 16:1	2	$[2M-H]^-$	6	985.6209	493.3168
GP	PC O-16:0	2	$[2M+HCOOH-H]^-$	5	1035.6680	495.3325
—	*nf*	4	—	—	1036.6720	—
GP	LPC 16:0	2	$[2M+HCOOH-H]^-$	1	1037.6760	496.3398
FA	FA 20:4;O4	2	$[3M-H]^-$	3	1103.6560	552.3298
—	*nf*	4	—	—	1104.6600	—

[glycan]-Cer, glycosphingolipids; CAR, acyl carnitine; CE, cholesteryl ester; Cer, ceramides; CL, cardiolipin; DG, diradylglycerolipid; FA, fatty acyl; FAHFA, fatty acyl esters of hydroxy fatty acid; GL, glycerolipid; Glc, glucose; GP, glycerophospholipid; HexCer, hexosylceramide; LPA, lysoglycerophosphate; LPC, lysoglycerophosphocholine; LPE, lysoglycerophosphoethanolamine; LPI, lysoglycerophosphoinositol; LPS, lysoglycerophosphoserine; LTC4, Leukotriene C4; Me; methyl branch; *nf*, not found; PA, glycerophosphate; PC, glycerophosphocholine; PE, glycerophosphoethanolamine; PG, glycerophosphoglycerol; PGP, glycerophosphoglycerophosphates; PI, glycerophosphoinositol; PS, glycerophosphoserine; SM, sphingomyelin; SP, sphingolipid; ST, sterol lipid; TG, triradylglycerolipid.

†Level of metabolite identification.

‡deriv, derivative.

Table S7. Mass spectrometry characterization with theorical (Th) and experimental (Exp) masses with the *m/z* and putative identification for the significant features of the SZ×CT comparison

Lipid Class	Putative identification	ID Level†	Adduct	Mass Error (ppm)	*m/z* Exp	*m/z* Th
—	*nf*	4	—	—	100.0728	—
—	*nf*	4	—	—	101.5198	—
—	*nf*	4	—	—	207.9167	—
GP	LPE 18:0	2	[M-H]-	4	480.3114	481.3168
ST	ST 26:1;O5	2	$[M+HCOOH-H]^-$	6	481.3145	436.3189
GP	LPS 19:0	2	$[M-H]^-$	4	538.3174	539.3223
ST	ST 18:4;O2	2	$[2M-H]^-$	8	539.3208	270.1620
GP	LPC 16:0	2	$[M+HCOOH-H]^-$	3	541.3365	496.3398
GP	LPC O-18:1	2	$[M+Cl]^-$	2	542.3393	507.3689
GP	LPA 24:0	2	$[M+Na-2H]^-$	2	543.3421	522.3685
GP	LPS 21:0	2	$[M-H]^-$	5	566.3490	567.3536
GP	LPC 18:1	2	$[M+HCOOH-H]^-$	3	567.3524	522.3560
GP	LPC 22:5	2	$[M+HCOOH-H]^-$	4	614.3486	569.3481
SP	Hex(5)-HexNAc(2)-Cer 42:2;O2	2	$[M-3H]^{3-}$	6	620.3373	1864.0445
SP	Hex(3)-HexNAc-KDN(2)-Cer 44:1;O2	2	$[M-3H]^{3-}$	1	621.3399	1867.0442
ST	CE 16:1	2	$[M+K-2H]^-$	7	659.5131	622.5689
SP	SM 32:1;O2	2	$[M-H]^-$	1	673.5293	674.5363
GP	PA O-40:4	2	$[M-H-H_2O]^-$	1	719.5374	738.5563
SP	HexCer 34:1;O2	2	$[M+Na-2H]^-$	2	720.5408	699.5649
SP	SM 32:0;O2	2	$[M+HCOOH-H]^-$	8	721.5444	331.2643
FA	FAHFA 24:3-(2-O-24:0)	2	$[M-H]^-$	8	727.6554	728.6683
SP	CL 72:0	2	$[M-2H]^{2-}$	5	731.5375	686.5363
—	*nf*	4	—	—	733.5537	698.5849
—	*nf*	4	—	—	734.5572	753.5672
SP	Cer 44:1;O2	2	$[M+CH_3COOH-H]^-$	5	736.6858	677.6686

SP	SM 40:1;O2	2	[M-H]-	1	785.6548	786.6615
GP	PE 40:7	2	[M-H]-	6	788.5286	789.5309
SP	SM 40:1;O2	2	[M+HCOOH-H]-	5	831.6636	786.6615
SP	HexCer 42:1;O2	2	[M+Na-2H]-	3	832.6672	811.6901
GL	TG 51:7	2	[M-H]-	4	833.6697	834.6737
SP	SM 41:1;O2	2	[M+HCOOH-H]-	4	845.6788	800.6771
SP	SM 41:1;O2	2	[M+HCOOH-H]-	2	846.6819	801.6850
ST	18:3-Glc-Campesterol	2	[M+HCOOH-H]-	6	867.6409	822.6374
SP	SM 42:1;O2	2	[M+CH_3COOH-H]-	3	873.7088	828.7084
SP	SM 42:1;O2	2	[M+CH_3COOH-H]-	1	874.7135	815.7006
GL	TG 54:7	2	[M-H]-	2	875.7151	876.7207
GP	PI O-39:2	2	[M-H]-	5	889.6218	890.6248
GP	PGP-Me	2	[M-H]-	0	899.6509	900.6584
GP	PE 46:4	2	[M+Na-2H]-	9	900.6545	879.6717
GP	PI O-40:2	2	[M-H]-	5	903.6377	904.6405
GP	PC O-40:4	2	[M-H+CH_3COONa]-	1	904.6418	823.6455
GL	TG 54:7	2	[M+K-2H]-	3	913.6670	876.7207
GP	PC 46:7	2	[M-H]-	6	914.6698	915.6717
GP	PS 43:2	2	[M+HCOOH-H]-	9	930.6524	885.6459
GP	PC O-16:0	2	[2M+HCOOH-H]-	5	1035.6680	495.3325
—	*nf*	4	—	—	1036.6720	—
GP	LPC 16:0	2	[2M+HCOOH-H]-	1	1037.6760	496.3398
FA	FA 20:4;O4	2	[3M-H]-	3	1103.6560	552.3298
—	*nf*	4	—	—	1104.6600	—

[glycan]-Cer, glycosphingolipids; CE, cholesteryl ester; Cer, ceramides; CL, cardiolipin; FA, fatty acyl; FAHFA, fatty acyl esters of hydroxy fatty acid; GL, glycerolipid; Glc, glucose; GP, glycerophospholipid; HexCer, hexosylceramide; LPA, lysoglycerophosphate; LPC, lysoglycerophosphocholine; LPE, lysoglycerophosphoethanolamine; LPS, lysoglycerophosphoserine; Me; methyl branch; *nf*, not found; PA, glycerophosphate; PC, glycerophosphocholine; PE, glycerophosphoethanolamine; PGP, glycerophosphoglycerophosphates; PI, glycerophosphoinositol; PS, glycerophosphoserine; SM, sphingomyelin; SP, sphingolipid; ST, sterol lipid; TG, triradylglycerolipid.

[†]Level of metabolite identification.

Table S8. Mass spectrometry characterization with theorical (Th) and experimental (Exp) masses with the *m/z* and putative identification for the significant features of the BD×SZ comparison

Lipid Class	Putative identification	ID Level†	Adduct	Mass Error (ppm)	*m/z* Exp	*m/z* Th
—	*nf*	4	—	—	101.5198	—
—	*nf*	4	—	—	207.9167	—
—	*nf*	4	—	—	214.9244	—
ST	ST 29:1;O	2	$[M+CH_3COOH-H]^-$	4	473.4018	428.4018
SP	Cer 42:2;O	2	$[M-H-H_2O]^-$	19	612.5961	631.6267
SP	Cer 44:2;O	2	$[M-H-H_2O]^-$	18	640.6277	659.6580
SP	SM 40:1;O2	2	$[M-H]^-$	1	785.6548	786.6615
SP	SM 41:1;O2	2	$[M-H]^-$	0	799.6701	800.6771
SP	SM 41:1;O2	2	$[M-H]^-$	6	800.6729	801.6850
SP	SM 40:1;O2	2	$[M+HCOOH-H]^-$	5	831.6636	786.6615
SP	HexCer 42:1;O2	2	$[M+Na-2H]^-$	3	832.6672	811.6901
GL	TG 51:7	2	$[M-H]^-$	4	833.6697	834.6737
SP	SM 41:1;O2	2	$[M+HCOOH-H]^-$	4	845.6788	800.6771
SP	SM 41:1;O2	2	$[M+HCOOH-H]^-$	2	846.6819	801.6850
ST	VIT D3 deriv‡	2	$[2M-H]^-$	4	859.6859	430.3447
SP	SM 41:1;O2	2	$[M+CH_3COOH-H]^-$	1	860.6979	815.7006
GL	TG 53:7	2	$[M-H]^-$	4	861.7015	862.7050
GP	PC O-44:3	2	$[M-H-H_2O]^-$	8	862.6984	881.7237
SP	SM 42:1;O2	2	$[M+CH_3COOH-H]^-$	3	873.7088	828.7084
SP	SM 42:1;O2	2	$[M+CH_3COOH-H]^-$	1	874.7135	828.7084
GL	TG 54:7	2	$[M-H]^-$	2	875.7151	815.7006
GP	PC O-44:5	2	$[M-H]^-$	1	876.6858	876.7207
GL	TG 54:7	2	$[M+K-2H]^-$	3	913.6670	876.7207

GP	PC(46:7)	2	[M-H]$^-$	6	914.6698	915.6717
—	*nf*	4	—	—	928.6860	—
FA	CAR 10:2	2	[3M-H]$^-$	2	932.6232	311.2097
SP	Cerebroside 3-sulphate	2	[M-H+HCOONa]$^-$	15	984.6292	917.6626
GP	LPC 16:1	2	[2M-H]$^-$	6	985.6209	493.3168

CAR, acyl carnitine; Cer, ceramides; FA, fatty acyl; GL, glycerolipid; GP, glycerophospholipid; HexCer, hexosylceramide; LPC, lysoglycerophosphocholine; *nf*, not found; PC, glycerophosphocholine; SM, sphingomyelin; SP, sphingolipid; ST, sterol lipid; TG, triradylglycerolipid.

†Level of metabolite identification.

‡deriv, derivative.

Tables S9 to S14. Available as .xls file.
